# Identifying major depressive disorder among US adults living alone using stacked ensemble machine learning algorithms

**DOI:** 10.3389/fpubh.2025.1472050

**Published:** 2025-02-21

**Authors:** Zhao Chen, Hao Liu, Yao Zhang, Fei Xing, Jiabao Jiang, Zhou Xiang, Xin Duan

**Affiliations:** ^1^Department of Orthopedic Surgery, Orthopedic Research Institute, West China Hospital, Sichuan University, Chengdu, China; ^2^Department of Emergency Management, Sichuan Center for Diseases Control and Prevention, Chengdu, China; ^3^Department of Pediatric Surgery, Orthopedic Research Institute, West China Hospital, Sichuan University, Chengdu, China; ^4^Department of Orthopedics Surgery, West China Sanya Hospital, Sichuan University, Sanya, Hainan, China; ^5^Department of Orthopedic Surgery, The Fifth People's Hospital of Sichuan Province, Chengdu, Sichuan, China

**Keywords:** major depressive disorder, adults living alone, stacked ensemble technique, machine learning, US NHANES

## Abstract

**Background:**

It has been increasingly recognized that adults living alone have a higher likelihood of developing Major Depressive Disorder (MDD) than those living with others. However, there is still no prediction model for MDD specifically designed for adults who live alone.

**Objective:**

This study aims to investigate the effectiveness of utilizing personal health data in combination with a stacked ensemble machine learning (SEML) technique to detect MDD among adults living alone, seeking to gain insights into the interaction between personal health data and MDD.

**Methods:**

Our data originated from the US National Health and Nutrition Examination Survey (NHANES) spanning 2007 to 2018. We finally selected a set of 30 easily accessible variables encompassing demographic profiles, lifestyle factors, and baseline health conditions. We constructed a SEML model for MDD detection, incorporating three conventional machine learning algorithms as base models and a Neural Network (NN) as the meta-model. Furthermore, SHapley Additive exPlanations (SHAP) analysis was used to explain the impact of each predictor on MDD.

**Results:**

The study included 2,642 adult participants who lived alone, of whom 10.6% (279 out of 2,642) had a PHQ-9 score of 10 or above, indicating the presence of MDD. The performance of our SEML model was robust, with an area under the curve (AUC) of 0.85. Further analysis using SHAP revealed positive correlations between the occurrence of MDD and factors such as sleep disorders, number of prescription medications, need for specific walking aids, leak urine during nonphysical activities, chronic bronchitis, and Healthy Eating Index (HEI) scores for sodium. Conversely, age, the Family Monthly Poverty Level Index (FMMPI), and HEI scores for added sugar showed negative correlations with MDD occurrence. Additionally, a U-shaped relationship was noted between the occurrence of MDD and both sleep duration and Body Mass Index (BMI), as well as HEI scores for dairy.

**Conclusion:**

The study has successfully developed a predictive model for MDD, specifically tailored for adults living alone using a stacked ensemble technique, enhancing the identification of MDD and its risk factors among adults living alone.

## Graphic abstract

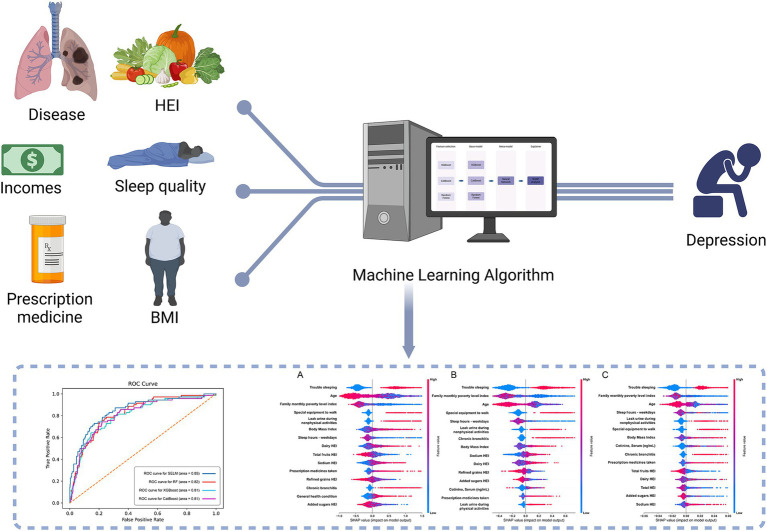

Schematic diagram of the study on identifying major depressive disorder among adults living alone using stacked ensemble machine learning algorithms.

## 1 Introduction

The past decade has witnessed a surge in the number of individuals living alone in the United States (U.S.), with a significant increase of 4.7 million to a total of 37.9 million from 2012 to 2022. This shift has garnered considerable attention due to the well-established link between living arrangements and mental health outcomes, particularly concerning Major Depressive Disorder (MDD). Recent research findings indicate a higher prevalence of MDD among adults living alone in the U.S. (6.4%) compared to those residing with others (4.1%) (1).

Major Depressive Disorder (MDD) is a prevalent mental health conditions marked by persistent mood lows, diminished interest, and a variety of affective, cognitive, somatic, and behavioral symptoms. These symptoms can profoundly impair psychosocial functioning and greatly diminish quality of life (2). The global prevalence of MDD has steadily risen over the past three decades, affecting approximately 5% of the adult population (3). It ranks as one of the leading causes of disability worldwide, contributing significantly to the overall burden of disease (4).

The therapy of MDD poses a challenge due to its heterogeneous nature (5). Key to improving patient prognosis is early detection and intervention. Unfortunately, stigma surrounding mental health assessments, inadequate mental health resources, and the common practice of concealing symptoms complicate the timely recognition and prediction of MDD (6). Although numerous studies have developed predictive models for MDD (6–10) to enhancing the likelihood of detection, there is currently a dearth of MDD predictive models specifically tailored for adults living alone.

Machine learning has demonstrated remarkable effectiveness in medical prediction (11), and is increasingly used in medical diagnostics (12). Machine learning models can adaptively learn from data to identify complex, nonlinear patterns (13). Furthermore, these models offer high interpretability, allowing researchers to understand model behavior and how decisions are made through various visualization and explanatory techniques (14, 15). Ensemble learning is a machine learning paradigm that enhances prediction accuracy by aggregating the predictions from several base models. It reduces the risk associated with individual models by combining their opinions, typically resulting in more accurate and robust predictions (16). The most common ensemble strategies include voting, averaging, stacking, and boosting. Among them, stacking has demonstrated strong predictive ability in tackling complex problems (17, 18). Nevertheless, to date, there is a dearth of research on the application of stacking in predicting the presence of MDD among adults living alone.

Therefore, we propose a stacked ensemble machine learning (SEML) model, utilizing data from the US National Health and Nutrition Examination Survey (NHANES), to predict MDD in adults living alone. We compared the performance of our SEML model with single machine learning models. Furthermore, we explored the interaction between predictors and the presence of MDD via the application of SHapley Additive exPlanations (SHAP) analysis. We aim to make a contribution to the field of MDD prediction in adults living alone and provide valuable insights for potential interventions and treatments.

## 2 Methods

### 2.1 Study design and participants

This cross-sectional study utilizes publicly available data from the US NHANES, an ongoing health-related initiative conducted periodically by the National Center for Health Statistics (NCHS) at the Centers for Disease Control and Prevention (CDC). The NCHS Ethics Review Board (ERB) approved the protocol for US NHANES, and all participants provided written informed consent (19).

Our study included data from respondents surveyed between 2007 and 2018. Living arrangements were evaluated by the number of people in the household, with only one person defined as living alone. Respondents under 18 years old, living with others, or with incomplete data were excluded.

### 2.2 Definition of MDD

MDD was evaluated using the Patient Health Questionnaire-9 (PHQ-9), which is based on the diagnostic criteria for MDD illustrated in the Diagnostic and Statistical Manual of Mental Disorders, Fourth Edition (DSM-IV). The PHQ-9 has been widely recognized for its accuracy in screening for MDD (20), with a score of 10 or higher indicating clinically significant symptoms (21). Therefore, in this study, we utilized a PHQ-9 score of 10 as the threshold for defining MDD.

### 2.3 Selection of predictors

The initial phase of the study included 72 feature variables related to demographic profiles, lifestyle factors, and baseline health conditions. Subsequently, a rigorous feature selection process was conducted, resulting in the identification of a subset of 30 variables for constructing the final predictive model. Within the initial pool of 72 variables, demographic characteristics encompassed participants’ age, gender, race, marital status, citizenship status, educational level, employment status, and income level. Lifestyle factors encompassed dietary quality, physical activity level, sleep patterns, and smoking habits. Dietary quality was assessed using the Healthy Eating Index-2020 (HEI-2020) (22), following the methodology proposed by Zhan et al. (23). Physical activity level was quantified by calculating participants’ weekly metabolic equivalent task (MET) minutes, derived from multiplying the MET values of specific physical activities by their respective weekly frequency and duration. The determination of the remaining variables relied on participants’ detailed responses to interview questions. Baseline health status was assessed based on participants’ medication usage and the presence of various diseases.

### 2.4 Statistical analysis

In our study, the raw data from the US NHANES database was used to construct machine learning models. Continuous variables were presented as means with standard deviations (SD), while categorical variables were displayed as counts and percentages. The statistical significance of differences in continuous and categorical variables was evaluated using independent t-tests and Chi-square tests, respectively, with a significance threshold set at a two-sided *p*-value of less than 0.05.

### 2.5 Model construction

**Figure 1:**
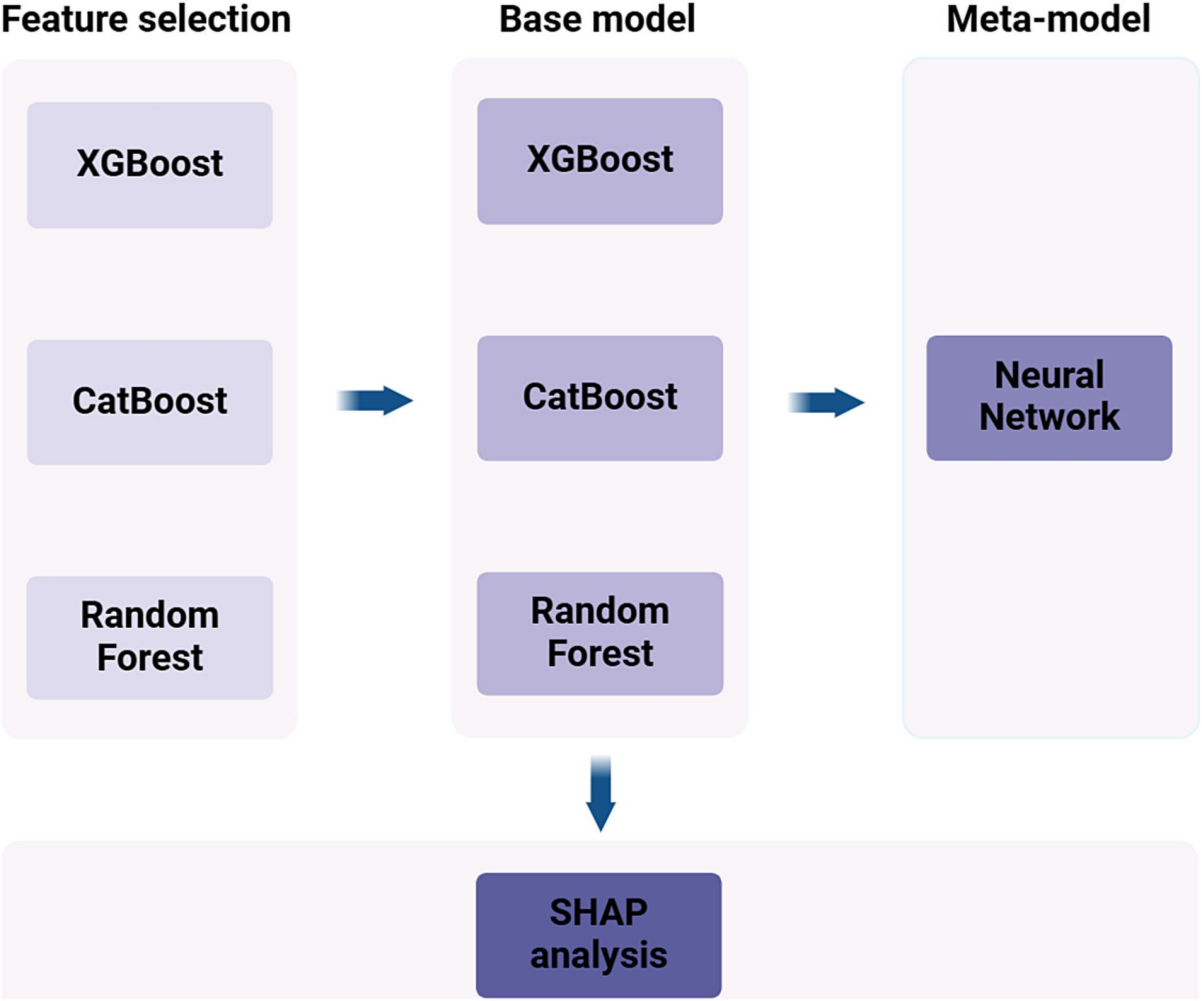
Flowchart of machine learning model construction.

In our study, a stratified sampling method was adopted to ensure an even distribution of MDD cases across different groups. The dataset was split into a training set, comprising 80% of the participants, and a test set, comprising the remaining 20%. To create the SEML model, we incorporated three algorithms as base models, including eXtreme Gradient Boosting (XGBoost), Categorical Boosting (CatBoost), and Random Forest (RF). Additionally, a Neural Network (NN) was designated as the meta-model (Figure 1). Both XGBoost and CatBoost belong to the Gradient Boosting Decision Trees (GBDT) algorithm, which employs ensemble learning to combine multiple decision trees using an additive model for predictions. RF represents another ensemble learning approach that enhances predictive power through voting or averaging the results of several decision trees. NN, a deep learning technique, process data by simulating the connections between biological neurons, making them adept at capturing complex nonlinear relationships. In this study, we integrated the aforementioned methods through SEML technique to fully leverage the strengths of each model and improve overall predictive performance.

Firstly, we employed the base models to select the top 30 variables in terms of importance out of the initial set of 72 variables. The feature importance was calculated by normalizing and summing the importance values from three different models. Both 10-fold cross-validation and Bayesian optimization were utilized by us for hyperparameter tuning and model evaluation (Details of the model hyperparameter settings are provided in the Supplementary materials). To enhance the predictive accuracy of the models, we performed normalization on the dataset. Additionally, the technique of Synthetic Minority Over-sampling Technique (SMOTE) was employed to mitigate class imbalance between MDD and non-MDD instances in the training data.

During the training phase, we utilized the predictions generated by each base model as input features for training the meta-model. When it came to the testing phase, the base models that had been trained were used to predict the test set. These predictions were then incorporated as input features to make the final prediction using the meta-model.

The assessment of model performance in this study employed the area under the Receiver Operating Characteristic (ROC) curve as the primary metric. Sensitivity, specificity, Youden index, and F1-score were also calculated at the optimal threshold to provide a comprehensive assessment. Furthermore, to elucidate the individual variables’ contributions and their importance in the model’s predictions, SHapley Additive exPlanations (SHAP) analysis was conducted.

All procedures were implemented in Python version 3.12.4.^1^

## 3 Study results and findings

### 3.1 Participant inclusion

**Figure 2:**
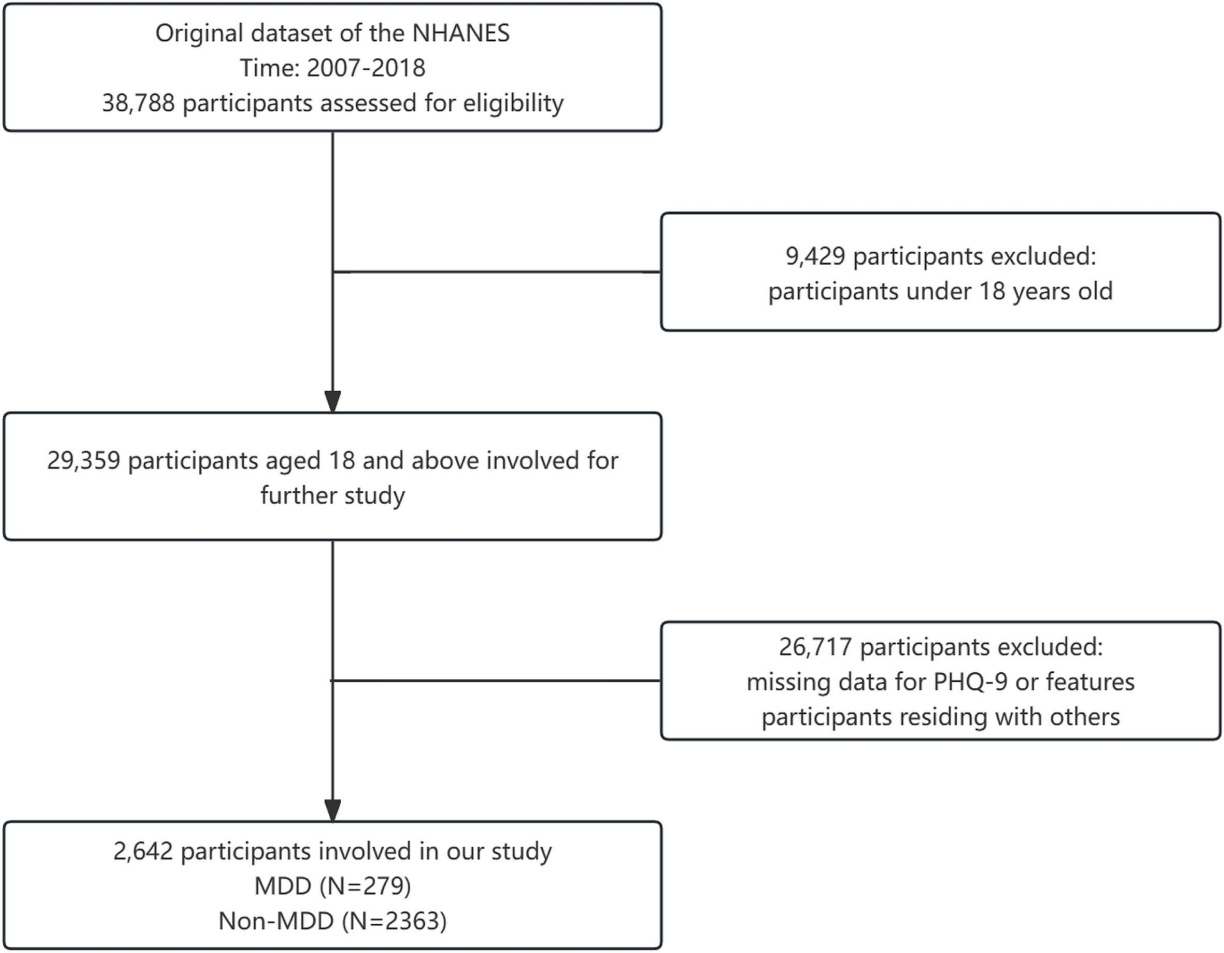
Flowchart of participants inclusion.

38,788 respondents were initially selected from the 2007–2018 US NHANES data. 9,429 participants under the age of 18 were excluded from the study. A further 26,717 participants were excluded due to incomplete data or residing with others. Finally, a total of 2,642 participants were included in the study (Figure 2).

### 3.2 General characteristics

Among these participants, 10.6% (279/2,642) of them had a PHQ-9 score of 10 or higher. No significant differences were observed in age (*p* = 0.2), marital status (*p* = 0.287), race (*p* = 0.644) and citizenship status (*p* = 0.315) between participants with and without MDD. Notable differences were found in gender (*p* < 0.001), education level (*p* < 0.001), employment status (p < 0.001) and family monthly poverty level index (*p* < 0.001; Table 1).

**Table 1:** Baseline characteristics according to depression or not.

	Non-depression (*N* = 2,363)	Depression (*N* = 279)	Total (*N* = 2,642)	*p*-value
Gender				
Male	1,057 (44.7%)	113 (40.5%)	1,170 (44.3%)	<0.001
Female	1,306 (55.3%)	166 (59.5%)	1,472 (55.7%)	
Age (year)				
Mean (SD)	60.2 (16.5)	56.4 (14.9)	59.8 (16.4)	0.2
Median [Min, Max]	63.0 [20.0, 80.0]	59.0 [20.0, 80.0]	63.0 [20.0, 80.0]	
Education level				
Less than 9th grade	133 (5.6%)	26 (9.3%)	159 (6.0%)	<0.001
9-11th grade	268 (11.3%)	50 (17.9%)	318 (12.0%)	
High school graduate/GED or equivalent	572 (24.2%)	59 (21.1%)	631 (23.9%)	
Some college or AA degree	735 (31.1%)	95 (34.1%)	830 (31.4%)	
College graduate or above	655 (27.7%)	49 (17.6%)	704 (26.6%)	
Marital status				
Married or Living with partner	75 (3.2%)	8 (2.9%)	83 (3.1%)	0.287
Widowed, divorced or separated	1,619 (68.5%)	204 (73.1%)	1823 (69.0%)	
Never married	669 (28.3%)	67 (24.0%)	736 (27.9%)	
Race				
Mexican American	209 (8.8%)	28 (10.0%)	237 (9.0%)	0.644
Other Hispanic	189 (8.0%)	27 (9.7%)	216 (8.2%)	
Non-Hispanic White	1,192 (50.4%)	135 (48.4%)	1,327 (50.2%)	
Non-Hispanic Black	598 (25.3%)	73 (26.2%)	671 (25.4%)	
Other Race - Including Multi-Racial	175 (7.4%)	16 (5.7%)	191 (7.2%)	
Citizenship status				
Citizen by birth or naturalization	145 (6.1%)	22 (7.9%)	167 (6.3%)	0.315
Not a citizen of the US	2,218 (93.9%)	257 (92.1%)	2,475 (93.7%)	
Employment status				
Employed	1,027 (43.5%)	81 (29.0%)	1,108 (41.9%)	<0.001
Unemployed	1,336 (56.5%)	198 (71.0%)	1,534 (58.1%)	
Family monthly poverty level index				
Mean (SD)	2.27 (1.51)	1.58 (1.34)	2.20 (1.51)	<0.001
Median [Min, Max]	1.81 [0, 5.00]	1.11 [0, 5.00]	1.69 [0, 5.00]	

**Figure 3:**
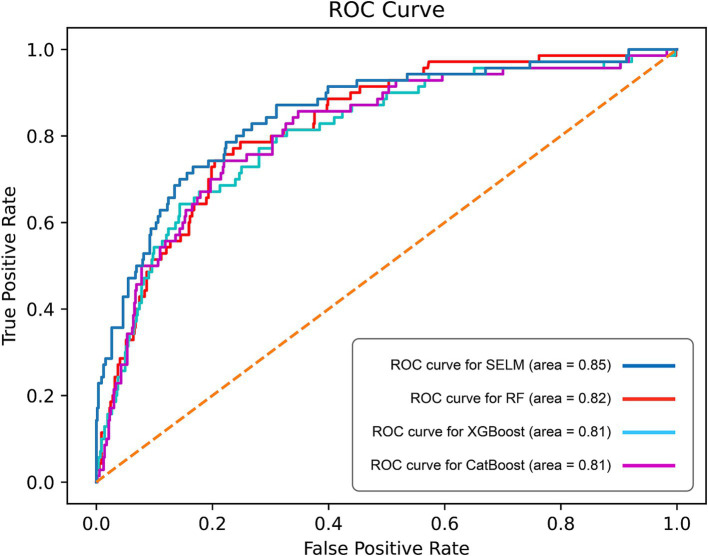
Curves of receiver operating characteristic (ROC) of different algorithms in test set. ROC, receiver operating characteristic; AUC, Area Under the Curve.

### 3.3 Included predictors

The final set of 30 feature variables integrated into the predictive model could be categorized as follows: demographic characteristics, including DMDMARTL, RIDAGEYR, IND235, and INDFMMPI; lifestyle factors, including HEI2020_ALL, HEI2020_DAIRY, HEI2020_ADDEDSUGAR, HEI2020_FATTYACID, HEI2020_FRT, HEI2020_GREENNBEAN, HEI2020_SODIUM, HEI2020_REFINEDGRAIN, HEI2020_SATFAT, HEI2020_SEAPLANTPRO, HEI2020_TOTALFRT, HEI2020_TOTALPRO, HEI2020_VEG and HEI2020_WHOLEGRAIN, LBXCOT, PAD680, TPA, SLD012, and SLQ050; Baseline health status, which encompassed BMXBMI, RXDDAYS, RXDCOUNT, PFQ054, MCQ160k, KIQ046, and HSD010. For more detailed information on each feature, please refer to Table 2.

**Table 2:** Explanation of the predictors used in this study.

Code	Variable type	Label	Involvement/Exclusion
BMXBMI	Continuous variables	Body Mass Index (kg/m**2)	Involvement
BPQ020	Categorical variables	Ever told you had high blood pressure	Exclusion
DIQ010	Categorical variables	Doctor told you have diabetes	Exclusion
DMDCITZN	Categorical variables	Citizenship status	Exclusion
DMDEDUC2	Categorical variables	Education level - Adults 20+	Exclusion
DMDMARTL	Categorical variables	Marital status	Involvement
HEI2020_ADDEDSUGAR	Continuous variables	Added sugars HEI	Involvement
HEI2020_ALL	Continuous variables	Total Healthy Eating Index (HEI)	Involvement
HEI2020_DAIRY	Continuous variables	Dairy HEI	Involvement
HEI2020_FATTYACID	Continuous variables	Fatty acids HEI	Involvement
HEI2020_FRT	Continuous variables	Whole fruits HEI	Involvement
HEI2020_GREENNBEAN	Continuous variables	Greens and beans HEI	Involvement
HEI2020_REFINEDGRAIN	Continuous variables	Refined grains HEI	Involvement
HEI2020_SATFAT	Continuous variables	Saturated fats HEI	Involvement
HEI2020_SEAPLANTPRO	Continuous variables	Seafood and plant proteins HEI	Involvement
HEI2020_SODIUM	Continuous variables	Sodium HEI	Involvement
HEI2020_TOTALFRT	Continuous variables	Total fruits HEI	Involvement
HEI2020_TOTALPRO	Continuous variables	Total protein foods HEI	Involvement
HEI2020_VEG	Continuous variables	Total vegetables HEI	Involvement
HEI2020_WHOLEGRAIN	Continuous variables	Whole grains HEI	Involvement
HSD010	Categorical variables	General health condition	Involvement
HSQ500	Categorical variables	SP have head cold or chest cold	Exclusion
HSQ510	Categorical variables	SP have stomach or intestinal illness?	Exclusion
HSQ520	Categorical variables	SP have flu, pneumonia, ear infection?	Exclusion
HSQ571	Categorical variables	SP donated blood in past 12 months?	Exclusion
IND235	Categorical variables	Monthly family income	Involvement
INDFMMPC	Categorical variables	Family monthly poverty level category	Exclusion
INDFMMPI	Continuous variables	Family monthly poverty level index	Involvement
INQ012	Categorical variables	Income from self-employment	Exclusion
INQ020	Categorical variables	Income from wages/salaries	Exclusion
INQ030	Categorical variables	Income from Social Security or RR	Exclusion
INQ060	Categorical variables	Income from other disability pension	Exclusion
INQ080	Categorical variables	Income from retirement/survivor pension	Exclusion
INQ090	Categorical variables	Income from Supplemental Security Income	Exclusion
INQ132	Categorical variables	Income from state/county cash assistance	Exclusion
INQ140	Categorical variables	Income from interest/dividends or rental	Exclusion
INQ150	Categorical variables	Income from other sources	Exclusion
KIQ022	Categorical variables	Ever told you had weak/failing kidneys	Exclusion
KIQ042	Categorical variables	Leak urine during physical activities	Exclusion
KIQ046	Categorical variables	Leak urine during nonphysical activities	Involvement
LBXCOT	Continuous variables	Cotinine, Serum (ng/mL)	Involvement
MCQ010	Categorical variables	Ever been told you have asthma	Exclusion
MCQ160a	Categorical variables	Doctor ever said you had arthritis	Exclusion
MCQ160b	Categorical variables	Ever told had congestive heart failure	Exclusion
MCQ160c	Categorical variables	Ever told you had coronary heart disease	Exclusion
MCQ160d	Categorical variables	Ever told you had angina/angina pectoris	Exclusion
MCQ160e	Categorical variables	Ever told you had heart attack	Exclusion
MCQ160f	Categorical variables	Ever told you had a stroke	Exclusion
MCQ160g	Categorical variables	Ever told you had emphysema	Exclusion
MCQ160k	Categorical variables	Ever told you had chronic bronchitis	Involvement
MCQ160l	Categorical variables	Ever told you had any liver condition	Exclusion
MCQ160m	Categorical variables	Ever told you had thyroid problem	Exclusion
MCQ220	Categorical variables	Ever told you had cancer or malignancy	Exclusion
MCQ300A	Categorical variables	Close relative had heart attack?	Exclusion
MCQ300B	Categorical variables	Close relative had asthma?	Exclusion
MCQ300C	Categorical variables	Close relative had diabetes?	Exclusion
OCQ150	Categorical variables	Type of work done last week	Exclusion
PAD680	Continuous variables	Minutes sedentary activity	Involvement
PFQ049	Categorical variables	Limitations keeping you from working	Exclusion
PFQ054	Categorical variables	Need special equipment to walk	Involvement
RIAGENDR	Categorical variables	Gender	Exclusion
RIDAGEYR	Continuous variables	Age in years at screening	Involvement
RIDRETH1	Categorical variables	Race/Hispanic origin	Exclusion
RPA	Continuous variables	Recreational physical activities	Exclusion
RXDCOUNT	Categorical variables	Number of prescription medicines taken	Involvement
RXDDAYS	Categorical variables	Number of days taken medicine	Involvement
RXDUSE	Categorical variables	Taken prescription medicine, past month	Exclusion
SLD012	Continuous variables	Sleep hours - weekdays or workdays	Involvement
SLQ050	Categorical variables	Ever told doctor had trouble sleeping?	Involvement
SMQ680	Categorical variables	Used tobacco/nicotine last 5 days?	Exclusion
SMS	Categorical variables	Smoking status	Exclusion
TPA	Continuous variables	Total physical activity	Involvement
WPA	Continuous variables	Work-related physical activity	Exclusion

HEI, Health Eating Index.

### 3.4 Performance of machine learning models

The ROC curve analysis revealed that SEML model performed the best among the evaluated models, as evidenced by an AUC value of 0.85 (95% CI 0.84–0.88), Followed by the RF model, which achieved an AUC of 0.82 (95% CI 0.80–0.83), while the AUC values for the CatBoost and XGBoost models were 0.81 (95% CI 0.78–0.84) and 0.81 (95% CI 0.77–0.85), respectively (Figure 3). Moreover, when considering the optimal threshold, the SEML model exhibited superior performance in terms of sensitivity, Youden index, and F1 score, with values of 0.79, 0.57, and 0.50, respectively. Furthermore, the SEML model demonstrated a specificity of 0.78, which was marginally lower than the specificity of the XGBoost model, recorded at 0.86. Detailed results were recorded in Table 3.

**Table 3:** Performance evaluation of machine learning algorithms.

	AUC	Sensitivity	Specificity	Youden index	F1-score
SEML	0.85	0.79	0.78	0.57	0.50
CatBoost	0.81	0.74	0.78	0.52	0.47
XGBoost	0.81	0.64	0.86	0.50	0.46
RF	0.82	0.74	0.80	0.54	0.45

AUC, Area Under the Curve; SEML, Stacking Ensemble Machine Learning; CatBoost, Categorical Boosting; XGBoost, eXtreme Gradient Boosting; RF, Random Forest.

### 3.5 Importance of predictive features

**Figure 4:**
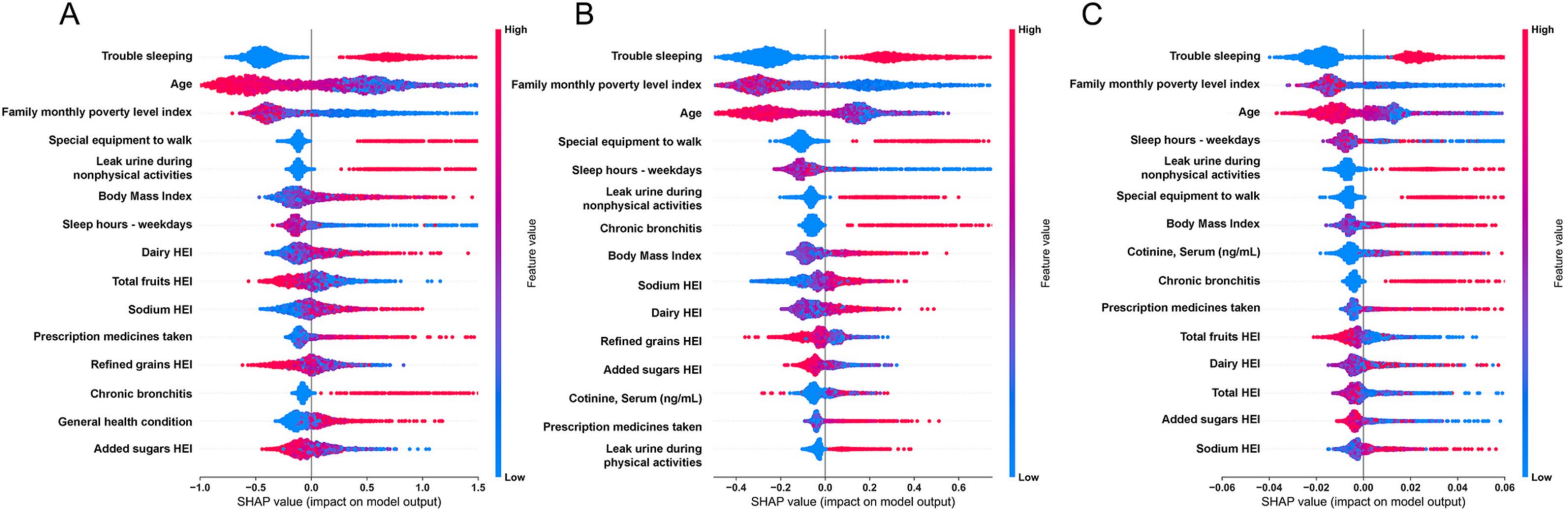
SHapley Additive exPlanations (SHAP) summary plot of features in the base models. **(A)** CatBoost; **(B)** XGBoost; **(C)** Random Forest.

In this study, we utilized SHAP analysis to determine the importance ranking of the features included in the base models. We summarized the top 15 features in terms of importance in the base models, and the detailed analysis results are presented in Figure 4. Across all three base models, the top 15 features consistently included SLQ050, SLD012, RIDAGEYR, INDFMMPI, BMXBMI, PFQ054, KIQ046, MCQ160K, HEI2020_SODIUM, HEI2020_DAIRY, and HEI2020_ADDSUGAR, amounting to a total of 12 features. Please refer to Table 3 for detailed interpretation regarding the code.

### 3.6 Features impact on MDD

**Figure 5:**
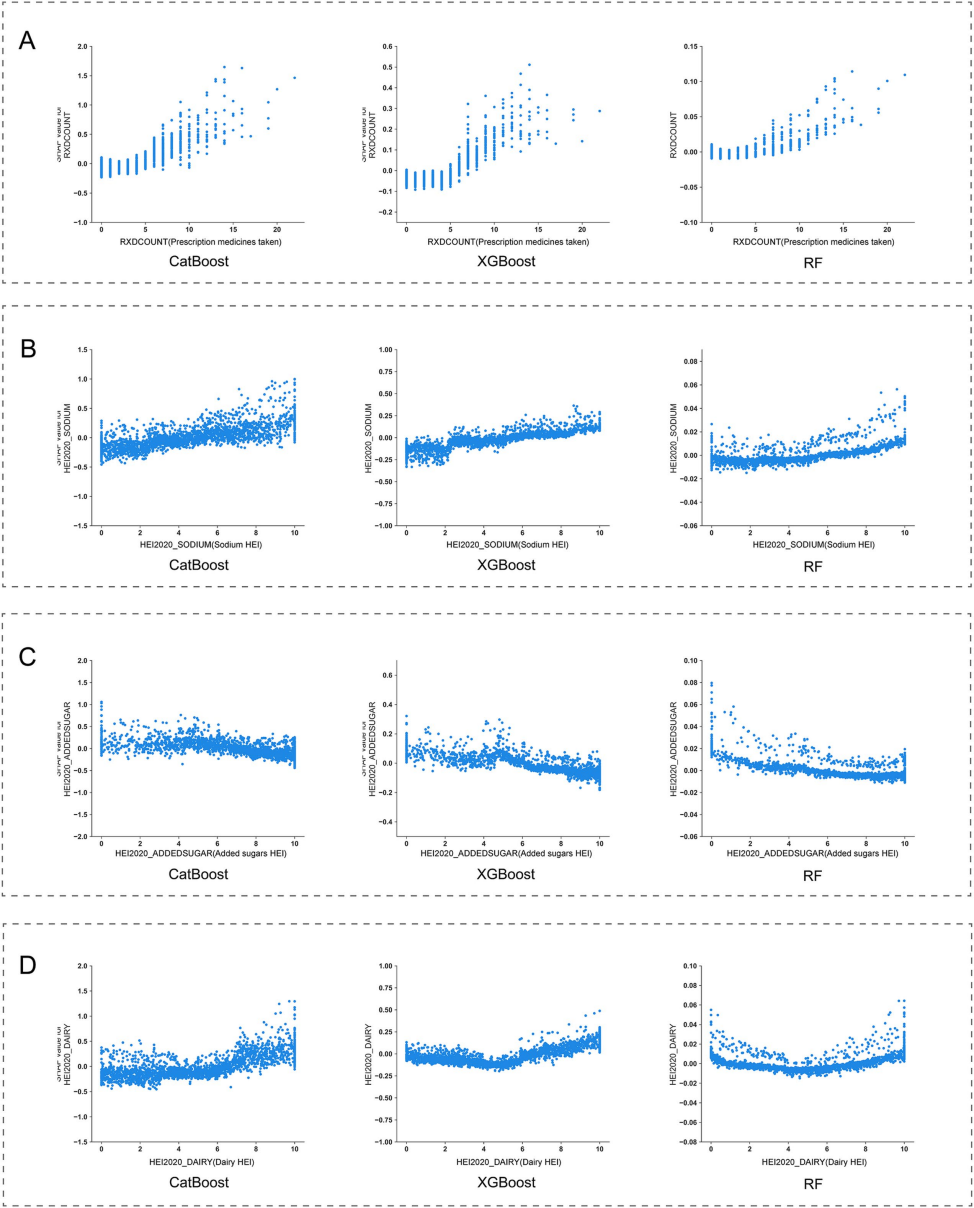
SHapley Additive exPlanations (SHAP) dependence plot of features in the base models. **(A)** RIDAGEYR; **(B)** BMXBMI; **(C)** SLD012; **(D)** INDFMMPI.

**Figure 6:**
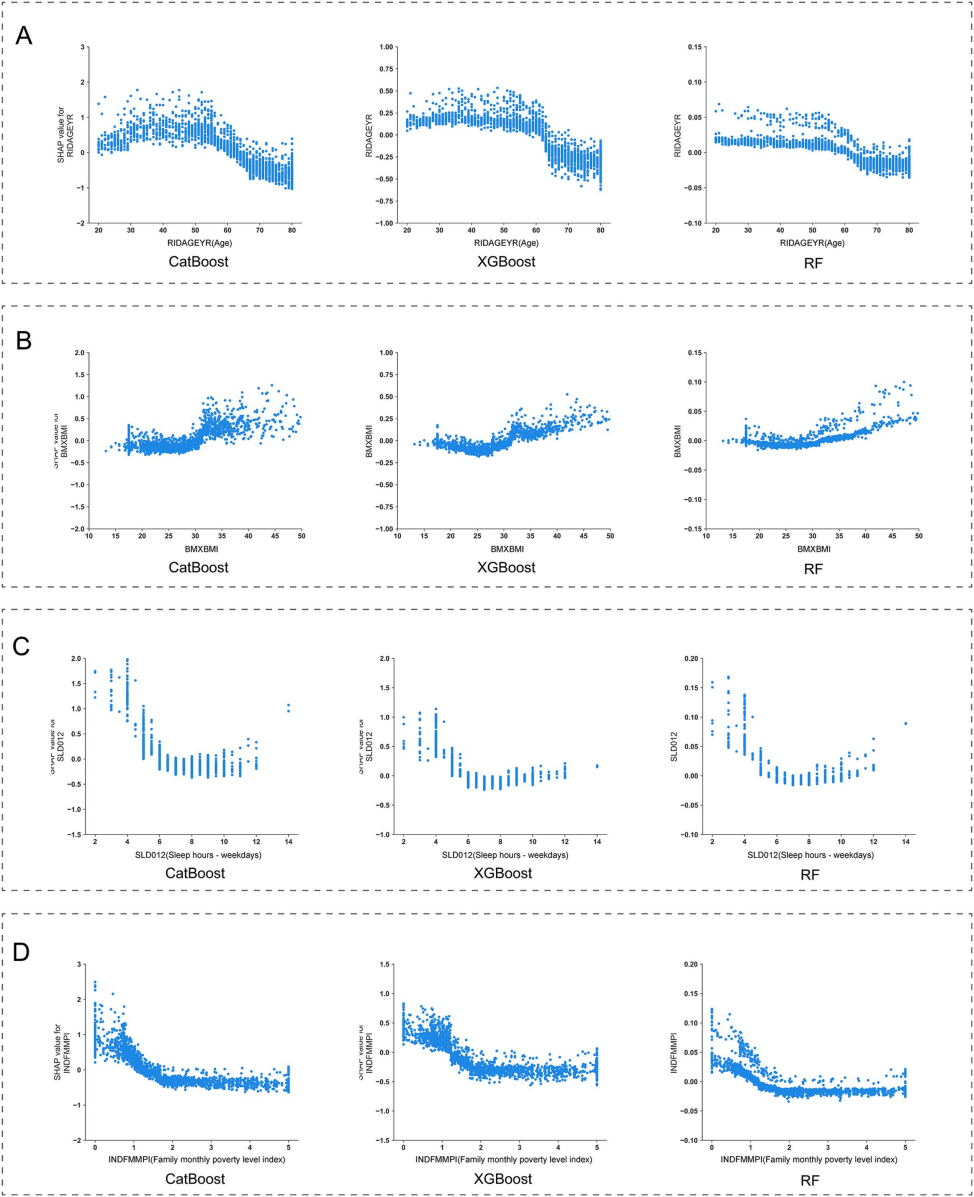
SHapley Additive exPlanations (SHAP) dependence plot of features in the base models. **(A)** RXDCOUNT; **(B)** HEI2020_SODIUM; **(C)** HEI2020_ADDEDSUGAR; **(D)** HEI2020_DAIRY.

By conducting SHAP analysis, we had effectively delineated the positive and negative impacts exerted by each feature on the occurrence of MDD. As illustrated in Figure 4, a set of features, namely SLQ050, PFQ054, KIQ046, BMXBMI, RXDCOUNT, HEI2020_DAIRY, HEI2020_SODIUM, MCQ160K, and HSD010 exhibited a positive correlation with the occurrence of MDD, whereas features such as RIDAGEYR, INDFMMPI, and HEI2020_ADDEDSUGAR manifested a negative correlation. Subsequent SHAP dependency analysis further corroborated these findings, as depicted in Figures 5, 6. Specifically, an augmentation in the values of features such as RXDCOUNT and HEI2020_SODIUM was concomitant with an escalated risk of developing MDD. Conversely, an escalation in the values of RIDAGEYR, INDFMMPI, and HEI2020_ADDEDSUGAR was linked to a diminished risk of MDD. Additionally, the dependence plot of SLQ012, BMXBMI, and HEI2020_DAIRY revealed a U-shaped relationship between their feature values and the occurrence of MDD. These findings aligned with the data presented in the SHAP summary plots, which depicted the influence of these features on MDD occurrence as a relatively complex interplay of both positive and negative impacts.

## 4 Discussion

Despite the existence of numerous studies utilizing machine learning methods to predict the occurrence of MDD (24–26), there remains a lack of research specifically focused on predicting MDD among adults living alone. Furthermore, many prediction models rely heavily on comprehensive clinical evaluations and laboratory data (9, 27), which limits their applicability. To address these limitations, this study is the first to focus on the population of adults living alone, utilizing easily accessible predictive variables from the US NHANES database to construct an MDD prediction model. This model incorporates 30 features related to demographic characteristics, lifestyle factors, and baseline health conditions. By employing a stacked ensemble technique, the model achieved an AUC value of 0.85, providing a new tool for predicting MDD in adults living alone.

To elucidate the contribution of each variable in the model’s predictions, we conducted SHAP analysis, a method based on game theory. The core idea is to calculate Shapley values to quantify each feature’s contribution to the prediction outcome, thereby enhancing the model’s transparency and interpretability. This helps researchers and decision-makers better understand the impact of different factors on the prediction results. Provides valuable insights for potential intervention and treatment strategies for MDD in adults living alone (28).

Compared to previous studies focused on the general adult population, our research identifies that, in addition to common influencing factors such as age, sleep, and income, daily sodium intake, added sugars, and dairy consumption are also significant factors affecting MDD in adults living alone. Furthermore, physical health factors such as mobility issues, urinary incontinence during non-physical activities, and chronic bronchitis also have important impacts on MDD (2, 27).

Firstly, within the demographic variables incorporated into our final predictive model, a significant correlation was observed between age and MDD occurrence. The SHAP dependence curve demonstrated a progressive decline in MDD risk with advancing age as participants aged 50 and above. Subsequently, as participants reached the age range of 60 to 65, we observed a shift in SHAP values from positive to negative, suggesting a potential protective effect of advanced age on the occurrence of MDD. This observation aligns with previous research by Villamil et al., who reported a significant reduction in MDD prevalence among women over 60 and men over 65 (29). Based on these findings, we can infer that individuals under 60 living alone face a higher risk of MDD compared to their older counterparts. Moreover, the Family Monthly Mean Poverty Index (FMMPI) of the participants emerged as a significant predictive feature. The SHAP dependence curve revealed a progressive decline in MDD risk as FMMPI increased within the range of 0 to 2. Previous studies have consistently demonstrated that lower income levels are typically associated with a heightened risk of MDD (30–32).Our findings further support this perspective, underscoring that adults living alone with a FMMPI below 2 are more vulnerable to MDD.

Regarding lifestyle characteristics, our findings underscore that both sleep quality and dietary quality are pivotal predictors of the occurrence of MDD. Prior study by Zhang et al. has identified sleep disorders as risk factors for secondary depression (33). Additionally, research by Baglioni et al. has shown that individuals with insomnia face a doubled risk of developing depression compared to those without sleep problems (34). Our current analysis employing SHAP reveals a positive correlation between sleep disorders and MDD occurrence, highlighting sleep disorders as significant risk factors for MDD among adults living alone. Another feature reflecting participants’ sleep status is sleep duration. In this study, the SHAP dependence curves displayed a U-shaped relationship between sleep duration and MDD occurrence. Notably, the lowest SHAP values are observed within a sleep duration of 7–8 h. This finding aligns with the conclusions drawn by Zhang et al., that individuals with an 8-h sleep duration exhibit the lowest risk of depression (35). Consequently, modifying sleep duration among adults living alone may potentially yield a substantial reduction in the risk of MDD occurrence. Regarding dietary quality, we employed the HEI scores to quantify the intake of various components. Through SHAP analysis, significant correlations were identified between the HEI scores for added sugar, dairy, and sodium components and the occurrence of MDD among adults living alone. Firstly, an inverse association was observed with the occurrence of MDD when HEI scores for added sugar exceeded 5. This finding aligns with prior research, which indicates that excessive intake of added sugars can have adverse effects on mental health and increase the risk of developing MDD (36, 37). Secondly, for sodium intake, a positive correlation was observed with the occurrence of MDD when HEI scores exceeded 2, indicating a potential link between low sodium diets and increased risk of MDD in adults living alone. This finding is consistent with findings from animal studies, which suggest that insufficient sodium intake may induce depressive symptoms, alleviated by sodium supplementation (38, 39). However, it is noteworthy that while these results from animal experiments exist, current limited human studies only support this inverse relationship between sodium intake and depression among females (40). Thus, the present findings still require further validation through larger, more rigorously designed studies. Lastly, regarding dairy intake, a U-shaped relationship was found between dairy intake and MDD occurrence. Specifically, the lowest contribution to MDD risk was observed when HEI scores reached 5, emphasizing that both excessive and insufficient dairy intake are closely associated with the occurrence of MDD in adults living alone. Given the current insufficiency of research on the relationship between dairy products and depression, particularly lacking longitudinal studies targeting the US population, we cannot yet determine a causal relationship between the two. These findings underscore the necessity for future research.

Moreover, this study has confirmed a series of baseline health conditions as key predictors for MDD. we systematically identified multiple health indicators closely associated with MDD occurrence. Firstly, body mass index (BMI), a common measure assessing whether an individual’s weight falls within a healthy range, shows a significant correlation with MDD risk. SHAP dependence curves indicate that elevated BMI (BMI > 25 kg/m^2^) is positively correlated with an increased risk of MDD. aligning with findings from previous epidemiological and clinical studies, which have shown that individuals who are overweight or obese have a higher risk of MDD (41–43). Conversely, a moderate BMI (18–25 kg/m^2^) appears to have a protective effect. This highlights the critical role of weight management in preventing MDD among adults living alone. Secondly, the number of prescription medications has also been identified as an important predictive feature. Generally, an increase in the number of prescription medications indicates potentially more complex health conditions among participants. In this study, we observed that the risk of MDD significantly rises when the number of prescription medications reaches five or more, indicating the need for special attention to mental health issues among adults living alone who require multiple medications. Additionally, the study found significant associations between the risk of MDD and the need for specific walking aids, symptoms of leak urine during nonphysical activities, and chronic bronchitis conditions. Changes in these participants’ physiological functions may indirectly increase the risk of MDD by affecting their quality of life. However, the causal relationships between these factors and MDD require further longitudinal research to be confirmed.

In summary, the findings presented above demonstrate that our model’s results are interpretable and meaningful. These outcomes not only corroborate the algorithm’s efficacy in predicting MDD but also offer a reference for the development of public health policies.

There are several limitations found in the current study. Firstly, the data utilized in this research was sourced exclusively from the US NHANES database, which contains information on the US population only. Therefore, caution ought to be applied when generalizing the predictive model to other countries. Secondly, the cross-sectional design of the study necessitates a high-quality prospective research to delve into the causal relationship between the identified predictors and the occurrence of MDD.

In conclusion, this study has successfully constructed a predictive model for MDD specifically tailored for adults living alone by applying stacked ensemble technique. Through SHAP analysis, the research thoroughly dissected the complex interplay among various predictors and MDD, providing a scientific reference for the development of personalized and effective intervention strategies.

## Data Availability

Publicly available datasets were analyzed in this study. This data can be found at: https://www.cdc.gov/nchs/nhanes.

## Footnotes

^1^ https://www.python.org/downloads/release/python-3124/
