# Supplementary material for: Identifying major depressive disorder among US adults living alone using stacked ensemble machine learning algorithms
Source: Front Public Health. 2025 Feb 21;13:1472050. doi: 10.3389/fpubh.2025.1472050 (PMC11892380; doi:10.3389/fpubh.2025.1472050)
Supplement: Supplementary file 1 [file Table_1.DOCX]

Supplementary Material

**Table S1.** The optimized parameters in models

| **SEML**  Activation=’sigmoid’, units=768, dropout=0.2, num_layers=5, Layer1: units=256, dropout=0.3; Layer2: units=640, dropout=0.3; Layer3: units=768, dropout=0.4; Layer4: units=768, dropout=0.3; Layer5: units=1024, dropout=0.3; L1 = 0.01, L2 = 0.01 |
| --- |
| **CatBoost**  learning_rate=0.035, depth=9, l2_leaf_reg=1.0, n_estimators=1000  **XGBoost**  Objective='binary:logistic', colsample_bytree=1.0, enable_categorical=False, eval_metric= 'logloss', learning_rate=0.1, max_depth=10, n_estimators=300, subsample=0.92, use_label_encoder=False  **Random forest**  Bootstrap=True, ccp_alpha=0.0, criterion='gini', max_depth=20, max_features='sqrt', min_impurity_decrease=0.0, min_samples_leaf=1, min_samples_split=2, min_weight_fraction_leaf=0.0, n_estimators=500, verbose=0 |
